# Obituary

**Published:** 1909-02-15

**Authors:** 


### OBITUARY.

### WILLIAM HENRY DORANCE, D.D.S.

Dr. Dorrance, for many years Professor of Prosthetic Dentistry in the University of Michigan, died at his home in Ann Arbor, Mich., Friday morning, January 22nd, 1909 after a lingering illness of many months. Dr. Dorrance was, born in Albion, N. Y., August 29th, 1842. His father was a jeweler, and while a boy in school, Dr. Dorrance learned the watchmaker and engravers trade. After completing his secondary schooling in the Albion Academy, he entered a dental office in the town and began the study of dentistry, but left it shortly to enter the army in 1861. He served three years under General McClellan as private and musician, but received his discharge in 1863, because of a serious illness. He settled in Jackson, Mich., where he began the practice of dentistry. In 1877 he was appointed demonstrator of mechanical dentistry in the University, and in 1879 he graduated from the dental department. In the same year he was made Professor of Prosthetic Dentistry and Metallurgy, which chair he held until 1902, when failing health caused him to resign the chair. Since that time his health has never been such as to enable him to do much work at his profession. He died from Brights disease.

Dr. Dorrance had a genial social nature and consequently many friends. He was a member of many benevolent and social organizations, a 32nd degree mason. For many years he was the leader of the choir in the Baptist Church of which he was a member. He was, in his earlier years, very fond of fishing, and had a choice collection of rods and reels. He also was one of the first to introduce lawn tennis on the University campus. He had a strong penchant for all kinds of mechanical instruments, and much of his money was spent in buying fine and expensive dental and mechanical instruments. His laboratory was filled with all kinds of instruments and appliances that he bought, hoping to make some good use of them in advancing the mechanical art of his profession. He was a very skillful man and all his prosthetic work was done with remarkable mechanical skill and art. He delighted to undertake difficult problems and had the greatest satisfaction in overcoming any difficult mechanical problem, and was often consulted by others than dentists in the solution of vexing mechanical problems. As a teacher he was greatly beloved, although he was not pre-eminent in his work, largely because he disliked pedantry and because he could not succeed in making ideal mechanics of all his class. He enjoyed and succeeded best when he could get a small number around him in the unconventional atmosphere of his laboratory, where he could enlist their sympathy and compel their co-operation in the construction of some interesting and difficult denture or appliance. Those who got to him in this way always secured an inspiration that led them into attempting better things. His greatest professional success was probably in the construction of obturators for cleft palate and other orthopedic appliances. To this work he gave much time. He also did excellent work in dental metallurgy. He was a great reader, but never wrote much except as contributions to dental societies of which he was a member. He was well known and thought of by the profession in his own state, but never took an active part in national affairs. He was married in 1867 to Clara E. Baldwin, who survives him. A son and daughter W. H. Dorrance, Jr., and Susan J. Fox, both married, are the only children living.

